# Preparation and Characterization of an Electrospun Whey Protein/Polycaprolactone Nanofiber Membrane for Chromium Removal from Water

**DOI:** 10.3390/nano12162744

**Published:** 2022-08-10

**Authors:** Laura Cristina Ramírez-Rodríguez, María Ximena Quintanilla-Carvajal, Didilia Ileana Mendoza-Castillo, Adrián Bonilla-Petriciolet, Carlos Jiménez-Junca

**Affiliations:** 1 Maestría en Diseño y Gestión de Procesos Facultad de Ingeniería, Campus Universitario Puente del Común, Universidad de la Sabana, Km. 7 Autopista Norte, Chia 25001, Colombia; 2 Agroindustrial Processes Research Group, Campus Universitario Puente del Común, Universidad de La Sabana, Km. 7 Autopista Norte, Chia 25001, Colombia; 3 CONACYT, Ciudad de México 03940, Mexico; 4 Departamento de Ingeniería Química, Instituto Tecnológico de Aguascalientes, Aguascalientes 20256, Mexico; 5 Bioprospecting Research Group, Campus Universitario Puente del Común, Universidad de La Sabana, Km. 7 Autopista Norte, Chia 25001, Colombia

**Keywords:** adsorption, chromium, electrospinning, hybrid membrane, nanofiber, polycaprolactone, whey

## Abstract

Chromium pollution represents a worldwide concern due to its high toxicity and bioaccumulation in organisms and ecosystems. An interesting material to remove metal ions from water is a whey-protein-based material elaborated by electrospinning, which is an emerging method to produce adsorbent membranes with diverse applications. The aim of this study was to prepare an adsorbent membrane of whey protein isolate (WPI) and polycaprolactone (PCL) by electrospinning to remove chromium ions from water. The adsorbent membrane was synthesized by a central composed design denaturing WPI using 2-Mercaptoethanol and mixing it with PCL to produce electrospun nanofibers. The adsorbent membrane was characterized by denaturation, Scanning Electron Microscope, Fourier-Transform Infrared Spectroscopy, Contact Angle, Thermogravimetric Analysis, and X-ray Photoelectron Spectrometry. The adsorption properties of this membrane were assessed in the removal of chromium. The removal performance of the membrane was enhanced by an increase in temperature showing an endothermic adsorption process. The adsorption process of chromium ions onto the nanofiber membrane followed the Sips adsorption isotherm, while the adsorption kinetics followed a pseudo-second kinetics where the maximum adsorption capacity was 31.0 mg/g at 30 °C and pH 2. This work provides a novel method to fabricate a hybrid membrane with amyloid-type fibrils of WPI and PCL, which is a promising adsorbent to remove heavy metal ions from water.

## 1. Introduction

Water contamination from toxic heavy metals is one of the most serious worldwide environmental problems due to the rapid growth of industrial development and non-restrictive legislation [1]. Heavy metals are not biodegradable, exhibit high toxicity, bioaccumulated and bio-magnified in the food chain thus threatening the health of humans and ecological systems. Chromium (Cr) has been recognized as a potential risk to human health and the environment due to its high toxicity, carcinogenicity, mutagenicity and it is commonly found in wastewater of many industries [2]. According to the US-EPA and WHO, the maximum concentration for total chromium in drinking water is 100 µg/L and 50 µg/L [1], respectively. Similarly, the US-EPA and the European Union stated that the amount of chromium discharged to surface water should be below 2 mg/L for total chromium and 50 µg/L for Cr(VI) [3].

The removal of heavy metal ions from water should be performed efficiently, easily, without waste generation (e.g., sludge) and at low cost, and this topic represents an important environmental issue globally [4]. To reach the regulatory limits for water quality, several methods have been used to remove heavy metal ions from water including chemical precipitation, coagulation, ion exchange, electrodialysis, and among others [5,6]. Nowadays, a common method used for wastewater treatment is membrane filtration due to its easy implementation in large-scale applications where nanofiltration and reverse osmosis are used to remove heavy metals. However, these methods are high pressure-driven that demand high energy consumption due to the narrow pore size distribution of the membrane, which also tends to foul [7]. Alternatively, adsorption can be used to remove heavy metals due to its high efficiency, simplicity, versatility, the availability of different adsorbents besides their regeneration and recyclability of them [8].

To overcome the high energy consumption and fouling of the membrane filtration process in the removal of heavy metal ions, the adsorptive membrane technology has been a better option due to its convenient operation using membranes with higher pore size, which combines the adsorption potential of the material and the filtration performance of the membrane [9]. In recent years, nanotechnology has been used in adsorbents production to obtain high-efficacy adsorbents for pollutant removal [10]. For example, membrane-based adsorption technologies composed of electrospun nanofibers have been receiving widespread attention due to the advantages of high efficiency, relatively simple production, versatility, large specific-surface area, high porosity, and good structural stability, which make it a promising technology for water treatment [11].

**Figure 1:**
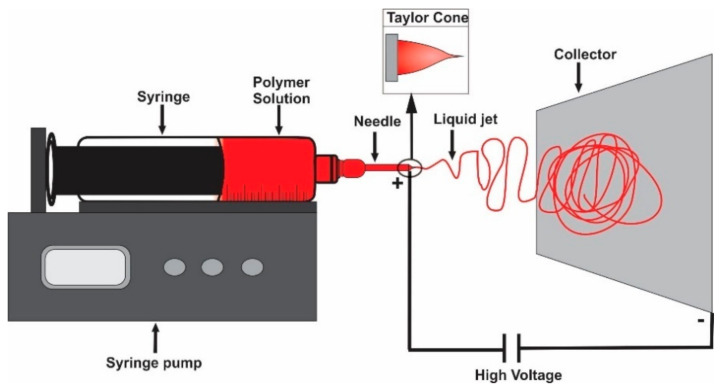
Illustration of an electrospinning apparatus.

Electrospinning is an emerging technique that has shown promising results because it is the simplest and most cost-effective method to produce a wide range of nanofibers in some cases smaller than 100 nm in diameter and is scalable for mass production [4]. To carry out the electrospinning process, several parameters must be considered such us polymer concentration, solution viscosity, electrical conductivity, and surface tension. Likewise, the operational parameters include electrical field strength, fluid flow rate and tip-to-collector distance [12]. When all conditions are met, electrostatic forces between the needle and the collector attract the solution, the Taylor cone is formed and finally the fibers are obtained (Figure 1).

Nanofibers produced by electrospinning for heavy metal removal have been gaining relevance due to their physical structure, good adsorption performance and favorable life cycle analysis [13]. Regarding chromium removal recently, Herath et al. elaborated polyacrylonitrile/ionic covalent organic framework hybrid nanofibers of 440 nm diameter by electrospinning to remove Cr(VI) from water, and the fibers presented an adsorption capacity of 173 mg/g [14]. Likewise, Sharafoddinzadeh et al. reported a maximum adsorption capacity of 225 mg/g to remove chromium from water using a polyacrylonitrile nanofibers of 165 nm of diameter made by electrospinning [15]. Ansari et al. prepared an electrospun zein/nylon-6 nanofiber membrane to remove Cr(VI) from water with an adsorption capacity of 4.73 mg/g [16].

Nanofibers elaborated by electrospinning are commonly made using polymers. Polycaprolactone (PCL) is widely used to fabricate nanofibers by electrospinning, because it bears high mechanical strength, easy processability, hydrophobicity and lack of toxicity [17]. PCL is known for its applications in tissue engineering owing to its biodegradability and biocompatibility, and has thus become a good choice for environmental applications due to its degradation time [18]. However, the use of PCL alone in adsorption materials is limited owing to its neutral charge and lack of functional groups [19]. Consequently, co-polymerization and blending with other materials have been recently employed in order to improve its chemical, physical and mechanical properties [20].

Blends of polymers with other materials have many advantages owing to the combination of properties and the strong economic incentives arising from their use [19]. Consequently, a blend with amyloids was proposed to improve the chemical limitations of PCL, which are proteins in a fibrillar state that can be formed under certain conditions when denatured [21]. Hence, amyloid fibrils have been widely used recently for environmental remediation especially in the recovery of heavy metal ions owing to the high affinity of amyloids with these pollutants [22]. Recently, Ramírez-Rodríguez et al. [23] developed an adsorptive amyloid membrane to remove heavy metals from wastewater using whey amyloid fibrils and activated carbon, which is an efficient and economic technology to remove a wide range of heavy metals. Similarly, numerous studies support the promising potential of amyloids to remove heavy metals from water [24,25,26]. Nevertheless, due to the mechanical and physical properties of amyloids, it is difficult to handle alone. For this reason, most studies use a support to hold amyloids into it [27]. In this way, PCL is an excellent material to make a hybrid adsorption membrane of PCL and amyloids of whey to enhance the advantages of both materials.

Whey is a by-product of the cheese industry composed mainly of β-Lactoglobulin (60%) and α-lactalbumin (20%), which is a promising raw material to produce amyloids due to its high content of protein [12]. Therefore, Ahmed et al. [19] studied PCL/WPC nanofibers for pharmaceutical applications, where it presented good morphology, wettability, high porosity and degradation ability. This is evidence that the synergy of the high metal binding capacity of proteins with a high surface area material may lead to promising electrospun membranes for heavy metal ion removal. The aim of this study was to produce WPI/PCL membranes by electrospinning to remove Cr ions from water. This approach is an opportunity to give an added value to whey, which is a residue of the cheese industry. The estimations indicate that nine kilograms of whey are produced per one kilogram of cheese, and nowadays it is readily available in the dairy industry [28]. Furthermore, the use of electrospinning is a highly innovative method to produce amyloid-based materials allowing a targeted design of membranes for toxic ion removal.

## 2. Materials and Methods

### 2.1. Materials

Poly(epsilon-caprolactone) (PCL, Mn = 70–90 kDa) was obtained from Sigma Aldrich (St. Louis, MI, USA), Whey Protein Isolate (WPI) was purchased from Davisco Foods International Inc. (Eden Prairie, MN, USA) and it was 97% protein of which approximately 69% was β-Lactoglobulin and 22% was α-lactalbumin, activated carbon was obtained from Sigma Aldrich (USA) with a particle size of 150 µm. The 2-Mercaptoethanol (βME, 98%) was purchased from Bio-Rad Laboratories (Mississauga, ON, Canada). Tetrahydrofuran (THF) and Dimethyl Formamide (DMF) were obtained from AppliChem (AppliChem, Darmstadt, Germany), which were analytic grade and used as received.

### 2.2. Preparation of the Adsorptive Membrane

Polymeric dispersions of WPI and PCL were prepared according to a Central Composited Design (CCD) as reported in Table 1, which evaluated different WPI and PCL proportions, βME concentration and the collector distance of the electrospinning. Solutions of PCL and WPI with βME were dissolved in THF and DMF (7:3 *v*/*v*) separately and stirred overnight at room temperature to ensure complete dissolution [19]. The solutions of PCL and WPI with βME were mixed and completed with THF/DMF (7:3 *v*/*v*) to reach the desired final concentration and stirred for 5 h. Finally, the activated carbon was added at a concentration of 0.05% to the solution with the best results to analyze if it produced a change on the adsorption capacity of fibrils.

A horizontal electrospinning Fluidnatek^®^ LE-10 (Bioinicia, Valencia, Spain) apparatus was used to produce the hybrid adsorbent membrane. The solution of WPI and PCL was pumped by a syringe pump operated at a flow rate of 4 mL/h, a voltage supply of 15 kV, collector distance from 7 to 13 cm [29]. The viscosity of the solutions was determined by Anton Paar MCR 502 rheometer (Anton Paar, Graz, Austria). The surface tension (ST) of the solutions was measured in the Sigma 700 tensiometer (Attension, Espoo, Finland) equipped with Wilhelmy plate at room temperature (19 °C) with 20 mL of sample, which was placed in a standard glass vessel to perform the measurement in triplicate.

The Design-Expert 10.0 software (Stat-Ease, Minneapolis, MN, USA) was used for the experimental design, response surface modeling, statistical regression analysis, process optimization and to evaluate the effect of the solution preparation on the viscosity, surface tension, fiber diameter and adsorption properties using a concentration of 100 mg/L of Cr(VI) at 30 °C, pH 3 for 24 h. The results were fitted via the response surface regression procedure using the following second-order polynomial Equation (1) and the models were evaluated following the criterion of desirability [30].

(1) $$Y\;=\;\beta_{0}\;+\;\sum_{i\;=\;1}^{n}\beta_{i}x_{i}\;+\;\sum_{i\;=\;1}^{n\;-\;1}\sum_{j\;=\;i\;+\;1}^{n}\beta_{ij}x_{i}x_{j}\;+\;\sum_{i\;=\;1}^{n}\beta_{ii}x_{i}{}^{2}$$

where *Y* is the predicted response, *β*_0_ is the regression coefficients, *β_i_* is the linear coefficient, *β_ii_* is the quadratic coefficients, *β_ij_* is the interaction coefficients and *X_i_* is the coded levels of independent variables, respectively.

### 2.3. Characterization of the Adsorptive Membrane

The characterization of the hybrid adsorbent membrane was carried out following the method proposed by Cong et al. [31]. The morphology of the hybrid membrane was analyzed by the fibril diameter using ImageJ and Tescan LYRA 3 Scanning Electron Microscopy (SEM) (TESCAN, Brno, Czech Republic) at an acceleration voltage of 4 kV [25], where all samples were gold-plated prior to observation. The surface chemistry of fibrils was determined by Fourier Transform Infrared Spectroscopy FTIR (Thermo Scientific^®^, Waltham, MA, USA) in the region of 400–1600 cm^−1^. The denaturation of WPI as amyloid was determined by Congo Red assay where aqueous solutions of 15% of WPI with different concentrations (0.1–1.2% *v*/*v*) of βME were analyzed following the method proposed by Yakupova et al. [32]. The effect of denatured WPI on PCL over thermal and mechanical performance was studied with thermogravimetric assay (TGA) on a TGA/DSC Mettler Toledo STAR 1 System (Mettler Toledo, Columbus, OH, USA) at a rate of 10 °C/min under nitrogen atmosphere in the range of 30–600 °C according to ASTM D6370. The contact angle was determined using distilled water at 20 °C with the sessile drop method in a Drop Shape Analysis System DSA (GH11, Krüss, Hamburg, Germany) according to ASTM-D7334-08 (2013). The average contact angle corresponded to three measurements for each hybrid membrane. X-ray Photoelectron Spectrometry (XPS) characterization was obtained on a Centeno-XPS/ISS/UPS X-ray Photoelectron Spectrometer (SPECS, Berlin, Germany) where the spectra were recorded using monochromatic Al Kα radiation (hv = 1486.6 eV), and the data analysis was performed with the CasaXPS program (Casa Software Ltd., Delray Beach, FL, USA).

### 2.4. Chromium Adsorption Capacity of the Adsorptive Membrane

To determine the adsorption capacity of the WPI/PCL hybrid membrane, the method of Sahebjamee et al. [33] was used where 20 g/L of the hybrid membrane was immersed in a heavy metal solution. Prior to the experiment, stock aqueous solutions of 1000 mg/L sodium chromate were adjusted to the desired pH using 0.1 M of HNO_3_. The adsorption experiments were carried out at batch conditions using 120 rpm and 30 °C. Kinetic studies were performed with different Cr concentrations from 50 to 100 mg/L, the adsorption data were fitted to kinetic (see Table S1) and isothermal classical models (see Table S2). Adsorption isotherms were obtained at different conditions of pH (2–5) and temperature (20 and 30 °C) with Cr concentrations from 10 to 300 mg/L using an equilibrium time of 24 h. Finally, the resulting solution was centrifugated at 6000 rpm, filtrated and analyzed by Atomic Absorption Spectrophotometer ContrAA 700 (Analytik Jena, Jena, Germany). The adsorption capacity (*qe*, mg/g) was calculated with the following Equation (2). Blank experiments were performed to ensure that no adsorption occurred on the walls of the apparatus used.

(2) $$qe\;=\;\frac{\left(C_{i}\;-\;C_{f}\right)*V}{m}$$

Thermodynamic parameters of Cr adsorption process were calculated using the Gibbs free energy (Δ*G*°, kJ/mol), see Equation (3). Standard enthalpy (ΔH°) and entropy (ΔS°) of hybrid membranes on Cr adsorption were calculated using the Van’t Hoff approach with Equation (4). Equilibrium adsorption constants were calculated following a standard procedure reported in literature.

(3) $$\Delta G\;=\;\;-\;RTlnK_{c}$$

(4) $$lnK_{c}\;=\;\frac{-\Delta H^{{}^{\circ}}}{RT}\;+\;\frac{\Delta S^{{}^{\circ}}}{R}\;$$

### 2.5. Statistical Analysis

All statistical analyses were completed using SPSS software version 17.0. Analysis of variance (ANOVA) was conducted to determine differences between treatments. Significant differences were established with Fisher’s least significant difference test with a significance level of 0.05. Experiments were performed in triplicate, and the results were reported as the mean and standard deviation of the measurements.

## 3. Results and Discussion

### 3.1. Preparation of the Adsorptive Membrane

To carry out the electrospinning process and to obtain the nanofibers with the desired properties, several parameters must be considered [34]. In this study, a CCD was established to investigate the effect of three influential factors: the proportion of WPI:PCL (%), the concentration of βME (%) and the collector distance (cm), which were used to obtain nanofibers with a thinner diameter (nm) and higher adsorption capacity (*qe*) for Cr(VI) removal (Table 2). Therefore, the solution properties such as viscosity and surface tension were considered since these properties are known to affect the electrostatic forces involved in the formation of the nanofibers [35,36].

The experimental design presented in Table 2 has 15 runs where the experimental results for the diameter, viscosity, surface tension, presence of beads and adsorption capacity are reported. It can be observed that the adsorption capacities, diameter, and viscosity varied strongly depending on the values of the influential factors. The experimental results of Table 2 were statistically analyzed by mean using ANOVA. Table 3 contains the fitting results of the second-order polynomial equation, the corresponding regression coefficients and the criterion of desirability. Statistical significance and accuracy of the models were confirmed via low *p*-value (<0.05), determination coefficient (R^2^) close to 1, and a high value (>0.05) of lack of fit. According to these metrics, all models were statistically significant at a 95% probability level (*p*-value < 0.05), the lack of fit was not statistically significant (*p*-value < 0.05) indicating that the models were adequate. All models fitted well to the experimental data due to all R^2^ values being over 0.94, which was according to the literature where it has been indicated that R^2^ value must be higher than 0.7 to obtain a good fit to the experimental data [37]. The adjusted R^2^ value for all models was above 0.8 thus confirming that the variations in the responses can be explained by the relationships obtained. Consequently, the models obtained in this study were useful to establish the best electrospinning preparation conditions of hybrid membranes of WPI and PCL for the Cr removal.

To obtain hybrid membranes of WPI-PCL with the best Cr removal properties, the diameter and adsorption capacity of the fibrils obtained by electrospinning were mainly evaluated. This study confirmed that the diameter and adsorption capacity were related due to the thinnest fiber diameter, which generated the highest value of adsorption capacity. This phenomenon was associated to a decrease in the diameter of the fibers leading to a membrane with a higher surface area [38,39]. Moreover, the diameter and *qe* were influenced significantly by the proportion of WPI:PCL (%), the concentration of βME (%) and the collector distance (cm). It was observed that the diameter decreased with the increase of the proportion of WPI (%) inversely proportional of PCL (%) and decrease in the concentration of βME (%). This can be explained via the composition effect on the viscosity and surface tension of the solution, where thinner fibers were obtained (experiments 12–14) using solutions with the lowest values of viscosity (0.005–0.007 Pa·s) and the intermediate values of surface tension (30.7–31.3 mN/m) were also obtained.

It was noted that a high proportion of WPI (90%) and low proportion of PCL (10%) were needed to obtain low viscosity values, and the concentration of βME (%) did not have a significant effect on the solution viscosity. Therefore, the solution viscosity was influenced mainly by the amount of PCL owing to its high molecular weight, which lead to a greater chain entanglement between PCL monomers and, consequently, an increment of the solution viscosity [40]. Furthermore, a range of viscosity values between 0.008 cP to 0.056 Pa·s has been reported in the literature as a starting point to obtain thin electrospinning fibrils without beads [41], where various authors have concluded that depending on the nature of the components of the spinning solution, the concentration and viscosity are essential to obtain structured fibers without defects such as beads [42]. In this study, the membrane with the highest adsorption capacity and thinner diameter was obtained at low viscosities, which agreed with other studies that used WPI and PEO, and reported the formation of fibers using low viscosity solutions with values ranging between 0.001 and 0.004 Pa·s.

Moreover, surface tension is another property involved in fibril formation that, in balance with the hydrostatic pressure and electrical forces, leads a controlled formation of the spinning jet producing fibers without defects [34]. Consequently, the results obtained by CCD demonstrated that the preparation of thin fibrils without beads requires a middle value (30 mN/m) of the surface tension, which was in agreement with the range of surface tension values of 19–70 mN/m reported by Ricaurte and Quintanilla Carvajal [41] to obtain thin nanofibers. To positively influence the surface tension according to CCD results, the proportion of WPI and PCL must be equilibrated and the concentrations of βME must be high (1.2%). Many authors stated that the surface tension of the solution can be influenced mainly by the solvent, which was true in this case as the surface tension of the DMF and THF were 35 and 28 mN/m and the surface tension of the solution of DMF/THF (3:7) was 30 mN/m [34,40,43]. Moreover, the entanglements of the polymer and the interaction with the WPI could modify the effect of solution surface tension, where WPI can behave as a surface-active agent [28] or it can decrease the surface tension [43] depending on the manner whey proteins interact with the polymer. Furthermore, the use of a reducing agent such as βME affects the surface tension of the solution as it induces the denaturation of whey proteins by reducing disulfide linkages changing some properties of the native proteins such as the surface tension [44].

In addition, the collector distance presented a significant effect on the diameter and the adsorption capacity for Cr removal on the hybrid membrane, where the collector distance must be 7 cm to obtain thin fibers with a high adsorption capacity. That is why, the high evaporation rate of the solvents allowed the formation of fibers at short distances, obtaining a high amount of thin fibers [45]. Moreover, it is important to find the distance between the tip and the collector to obtain fibers with a balance among the electrostatic forces and the solution properties, to prevent the formation of beads [42]. Drosou et al. [12] studied the effect of the collector distance to synthesize fibers of WPI and pullulan. These authors concluded that the fibers presented more bead defects with an increase in the collector distance. Thus, a decrease in the electrostatic field and jet splitting was obtained with an increment in the collector distance and using a fixed voltage.

The results obtained by the CCD were used to establish the best preparation conditions using the criterion of desirability (Table 4) with the aim of obtaining the fibers by electrospinning with the highest adsorption capacity and the lowest diameter.

The results showed that the adsorption capacity was favored at high amounts of WPI (~90%), βME (~1%) and a short distance (~7 cm). These results demonstrated that high amounts of WPI and βME displayed the best results. This is because whey proteins are rich in functional groups, which in this case, have been desaturated with βME. Comparing WPI with PCL alone, it lacks functional groups and, in consequence, it does not have the potential to remove heavy metals by itself. Moreover, a short distance promoted the elaboration of ultrathin nanofibers as discussed before.

Whey proteins have been studied owing to their high nutritional value and some authors have studied their interactions with metal ions because of their capacity to serve as carriers for metal complexes [46]. Whey protein is composed mainly of β-Lactoglobulin and α-Lactalbumin, where β-lactoglobulin is well known for its interaction with metal ions due to its free cysteine group (Cys-121) that acts as a binding site for metal ions of the d-block such as copper, silver and mercury [24]. Likewise, α-lactalbumin has a structural affinity for metal ions, which may replace the calcium site of the protein. Furthermore, the interaction between proteins and metal ions results in metalloproteins and metallocomplexes, where metalloproteins are formed by coordination bonds between metal ions and functional groups of amino acids, such as carboxyl [47]. Metallocomplex is an artificial system that lead to a binding of metal ions with proteins that interact through weak interactions such as hydrogen bonding, electrostatic, Wan der Waals forces, or donor–acceptor bonds. This binding arises via adsorption or intraparticle diffusion, where the metal ion of a metalloprotein is immersed in a protein structure forming a natural system such as hemoglobin [48].

In this study, the addition of βME was essential to promote the adsorption of Cr into the membrane, which without the addition of this reductant agent, the resulted membrane did not adsorb any metal ion. βME is an agent that is used to reduce the disulfide bonds of proteins leading to a tautomerization and breaking up of the protein’s quaternary structure, where an excess of βME holds the thiol groups of proteins in their reduced state [49]. Nguyen et al. studied the effect of βME on the denaturation of whey proteins and concluded that thiol reagents such as βME initiate thiol–disulfide exchange reactions with the disulfide bonds on α-lactalbumin and β-Lactoglobulin that lead to irreversibly denatured proteins [50]. Moreover, the presence of coordination bonds is desired to promote metal–protein interactions, which allows the metal to be inserted into the structure of the protein. For example, the free thiol group of the cysteine of β-Lactoglobulin has to be deprotonated for involvement in the metal coordination as well as tyrosine which can be deprotonated to produce a phenolate oxygen donor atom that can act as a ligand for metals of the d-block [47]. βME is quite a strong hydrogen-bond acceptor, capable of coordinating with many metal ions such as As, Ni, Pb and Zn [51]. The results demonstrated that βME is a critical factor in the adsorption capacity of the membrane, which denaturized the whey proteins of the WPI making it suitable for heavy metal removal. Furthermore, it was demonstrated the βME did not have an effect on the PCL at the evaluated conditions because fibrils produced with PCL alone and PCL with the higher concentration of βME evaluated did not present Cr removal.

#### Effect of the Total Solids and Activated Carbon Addition on the Preparation of the Membrane

**Figure 2:**
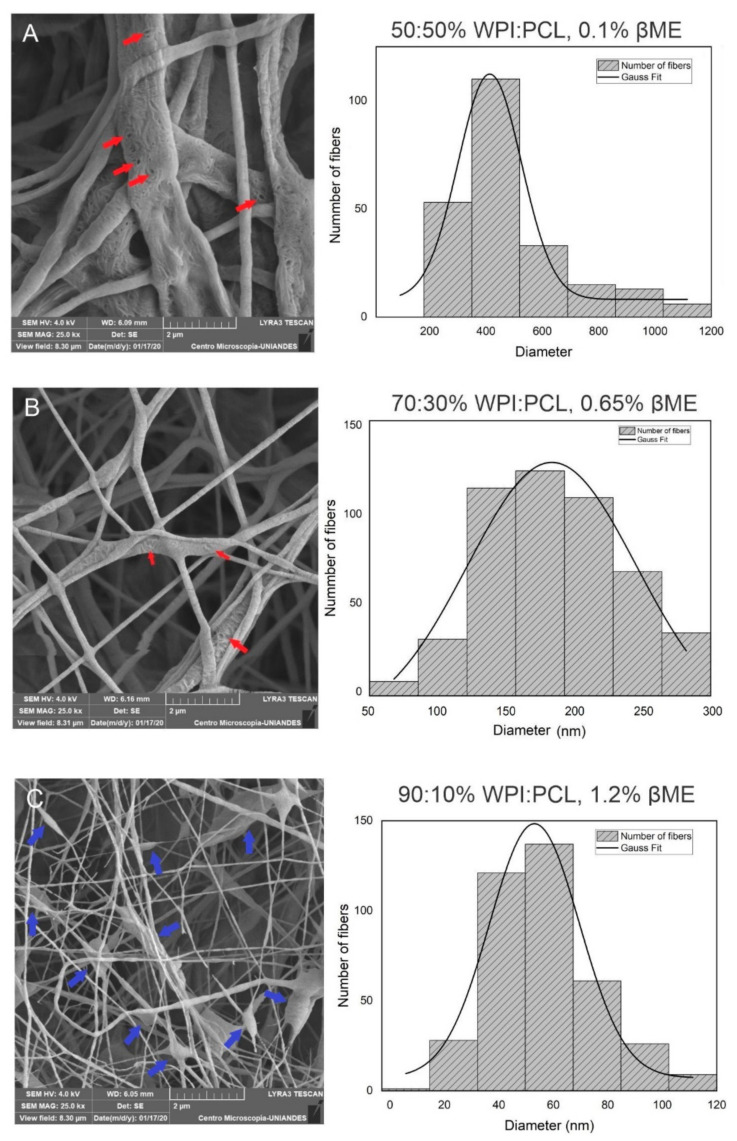

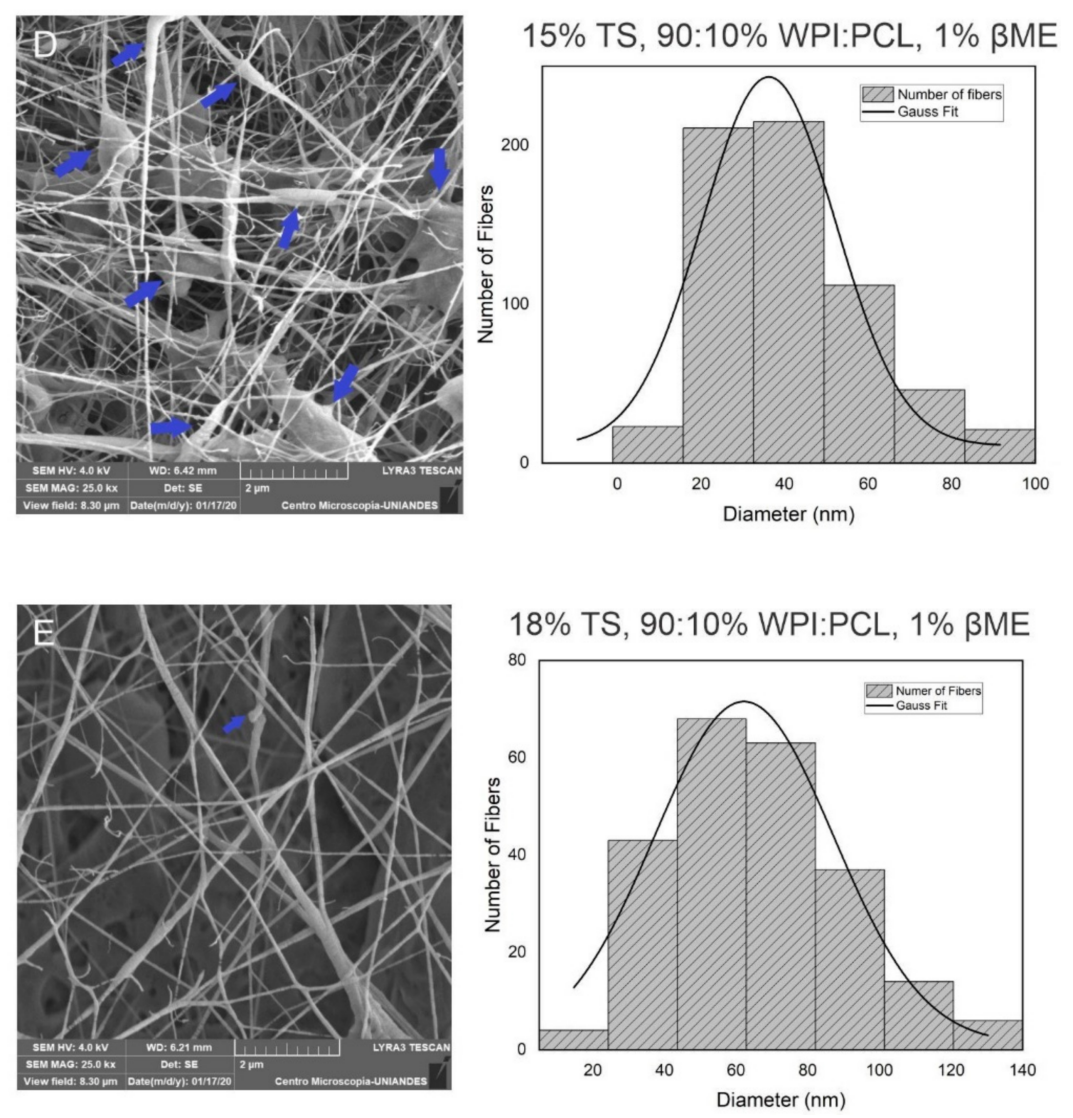
Morphological analysis of hybrid membrane of WPF and PCL by SEM. These results demonstrated the diameter change with the composition of % WPI:PCL and % βME at 7 cm of collector distance. (**A**) 50:50%, 0.1%; (**B**) 70:30%, 0.65%; (**C**) 90:10%, 1.2%; (**D**) membrane at the best conditions 15% of total solids, 90:10%, 1% (**E**) 18% of total solids, 90:10%, 1%. Graphical arrows in red represent the micropores in the fibers and blue arrows represent beads.

The membrane obtained by the CCD was achieved using 15% of total solids (TS), ~90% of WPI, ~1% βME and a collector distance of ~7 cm, which corresponded to the conditions that generated the best adsorption capacity and the thinner diameter (Table 4). The presence of beads was observed in the fibrils of the membrane (see Figure 2D). Note that it is well known that the properties of the spun solution affect the morphology of the fibers [41]. To overcome the presence of beads, this study considered that the concentration of the spun solution affected the stretching of the charged jet. Because there must be enough entangled polymer chains to form fibers that can reach the collector and, if this does not occur, the entangled polymer chains will break and the fragments can cause the formation of beads or beaded nanofibers [42]. For this reason, this study evaluated the effect of the content of the total solids ranging from 15% to 21% on the shape of the fibers (Table 5) to improve their shape and avoid bead formation.

The results demonstrated (see Table 5) that the fibers obtained using 18% of total solids on the solution presented the best results in terms of the adsorption capacity and there were no beads on the fibers. The adsorption capacity of these samples was improved by 3.6 times for Cr removal in comparison to the fibers prepared using 15%. Moreover, it was very difficult to obtain fibers using 21% of total solids due to the properties of the solution such as its high viscosity which did not allow the electrospinning process to occur. The enhancement of the adsorption capacity of the membrane prepared with a solution with 18% of solids was greater than the membrane made with 15% of solids due to the absence of beads on the fibers, which led a material almost without defects and with a homogeneous surface area promoting a better affinity between the surface and heavy metal. In contrast, the membrane obtained with a solution with 18% of total solids demonstrated fibers with a thicker diameter, without beads and an equilibrated surface area [52].

### 3.2. Characterization of Membrane

#### 3.2.1. SEM Characterization

To understand the morphology of the fibers obtained by the CCD, SEM characterization was used. The fiber diameter was analyzed varying the composition of PCL and WPI in the fiber solution at a collector distance of 7 cm. The results confirmed a fiber morphology in all samples and showed that thick fibers with a diameter of 413 nm were obtained using a solution with a high concentration of PCL at a proportion of 50:50% WPI:PCL (see Figure 2A). Decreasing the amount of PCL in the solution to 70:30% (Figure 2B) and 90:10% WPI:PCL (Figure 2C), the diameter of the fibers decreased obtaining diameter values of 160 and 53 nm, which represented an increase on the surface area [53]. However, the presence of beads increased by increasing the proportion of WPI in the spun solution where the same phenomenon was observed by Drosou et al. [12]. These authors elaborated electrospinning fibrils using a composite pullulan and WPI and noted that the fibrils presented beads using WPI above 70%.

As discussed, the viscosity, conductivity and surface tension of the spun solution are critical parameters that are affected by the polymer concentration, which also have an effect on the fiber diameter and the presence of beads in the fiber [41]. In this case, the polymer proportion on the fiber had a significant effect on the diameter of the fibers due to the amount and length of the polymer chain represented by the polymer weight that determines the amount of the polymer entanglement [54]. Thus, when the chain entanglements of polymer are high, the entanglements between the polymer and WPI within the solution maintain the jet formation during electrospinning process and bead-free fibers are obtained [43].

On the other hand, the diameter and presence of beads in the fibers were influenced by the concentration of βME in the spun solution. Note that the viscosity of the solution is modulated with the addition of βME owing to βME-induced unfolding of the globular proteins of WPI by the polypeptide chain entanglement and chain–chain interaction with the PCL. This enhanced the elasticity of the solution jet during the electrospinning process [28]. The same result was observed by Kabay et al. [55], who modified the viscosity of a BSA protein solution with the addition of βME and it was possible to spin the protein solution without the addition of a polymer, which would not have been possible with the protein alone.

The presence of some micropores was observed on the surface of the nanofibers obtained as shown in the Figure 2A,B. This result means that the membrane, besides having ultra-thin fibers that increase the surface area of the material, contains some micropores that also increase the surface area favoring the adsorption process. This is because a fiber with a smaller diameter decreases the particle size, which means that the surface area per volume increases and more fibers can occupy the same volume [38,39]. Analyzing the increase of a total solids concentration in the spun solution, Figure 2D showed that the solution with 18% of solids did not present beads into the fibers in comparison to the fibers obtained with the spun solution of 15% (see Figure 2C), which showed the presence of beads in the fibers. Thus, the formation of beads on the solution of 15% of solids was due to the low concentration of WPI and PCL and, in consequence, the solution viscosity was low. As stated, it is well known that viscosity is a critical factor in the formation of well-defined fibers because this property is related to polymer chain entanglements in the solution produced by the change of the polymer and solvent composition [39] which, in consequence, affect the viscoelastic response, charge relaxation times, and solvent evaporation rate [56]. Thus, continuous and homogenous fibers were obtained with an equilibrium of the composition, viscosity, conductivity, surface tension as well as process parameters, such as voltage, feed flow rate, and tip–collector distance [41].

#### 3.2.2. FT-IR

**Figure 3:**
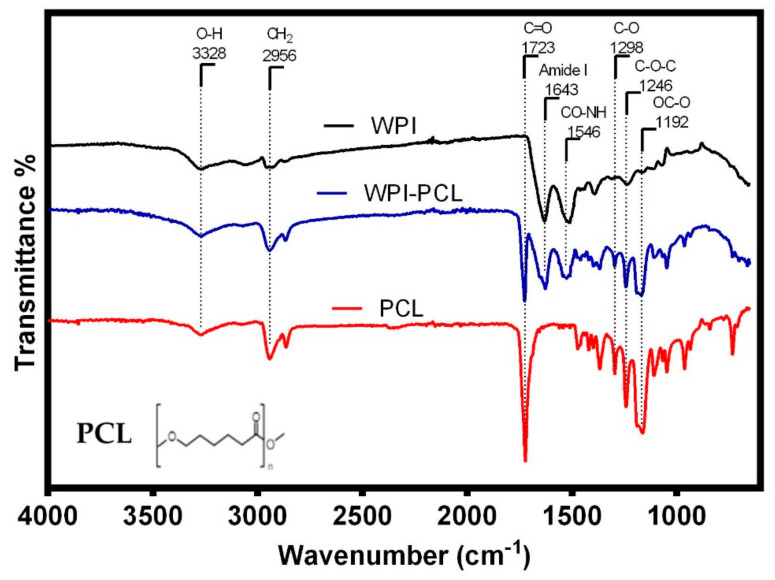
FTIR spectra of the hybrid membrane of WPI:PCL (90:10, blue line), WPI (black line) and PCL (red line).

The FT-IR analysis was used to identify the functional groups of the adsorptive membrane compared with their raw materials such as WPI and PCL to perform a qualitative characterization of the membrane composition. The FT-IR spectra (see Figure 3) of all samples showed a band at 3328 cm^−1^, which corresponded to stretching vibrations of -OH due to the presence of water [57]. The spectra of WPI and membrane showed characteristic bands of the peptide bonds of whey proteins at 1643 cm^−1^ related to a primary amide group (C=O, C-N) [43], and the band at 1546 cm^−1^ representing the secondary amide group (N-H, C-N) [58]. This demonstrated that the membrane contained the main functional groups of the WPI indicating that the fibers were mainly composed of WPI, which was the component with an affinity to heavy metals.

The spectra of PCL alone and the membrane presented the characteristic PCL band of the carbonyl stretching vibration at 1723 cm^−1^ [59], the bands at 2956 and 2907 cm^−1^ represented the symmetric and asymmetric stretching vibration of a methylene group (CH_2_) [17], the absorption bands at 1298 (C-O bands tension), 1246 (C-O-C symmetrical tension) and 1192 cm^−1^ (OC-O stretching) were associated with the C-O stretching vibrations on the crystalline phase of PCL [60]. The analysis of the FT-IR spectra showed that the membrane of PCL and WPI presented the characteristic bands of the functional groups of both components, thus demonstrating the successful blending of the components within the spun fibers. A similar result was obtained by Ahmed et al. [19] for the case of fibrils of whey protein and PCL for the antibiotic release. These authors confirmed a successful doping of whey protein in the fibrils, which proved that the electrospinning process only mixed both components and did not change the chemical structure of PCL and whey protein.

Furthermore, the results suggested the βME did not influence the chemical structure of the PCL and there was no appreciable difference between the spectra of WPI alone and the WPI in the electrospun fibers. Moreover, the used organic solvents did not have an effect on whey protein, similar to the study reported by Ahmed et al. [19].

#### 3.2.3. Congo Red Assay

**Figure 4:**
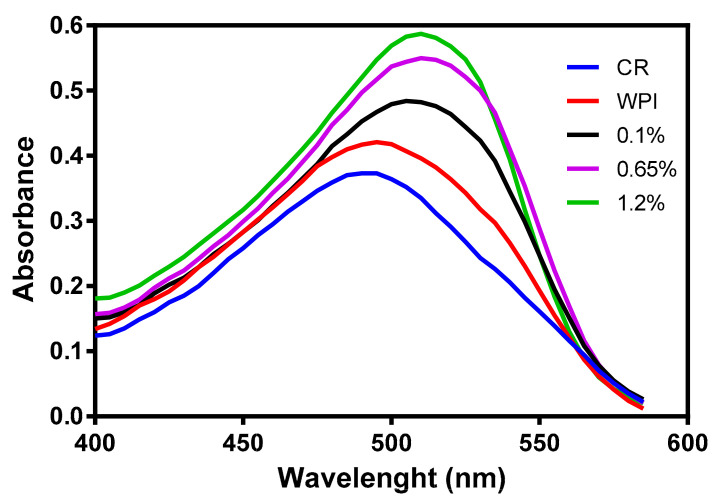
Congo red absorption spectra. Blue line represents red Congo dye as reference, red line represents an aqueous solution of WPI at 15% without βME, black, purple and green lines represent WPI in aqueous solution with different compositions of βME.

Congo Red (CR) assay was used to qualitatively identify amyloids and to determine the effect of the addition of βME on WPI that is a denaturant agent of proteins. CR is a specific dye that binds with the crossed-β-pleated sheet structure common to a variety of amyloid fibrils, thus causing an increase in the absorption from 490 nm and when bonded to a native protein to 510 nm when CR is bonded to amyloids [61]. CR analysis (see Figure 4) showed the absorption spectra of this dye alone and in solution with a WPI (14%) and presented a maximum absorption peak at 490 nm. The solution of WPI (14%) treated with different concentrations of βME showed an increase in the absorption band to 510 nm demonstrating the presence of amyloid structures in the solution. Moreover, there was an increment of the absorbance at 510 nm when the concentration of βME increased in the solution of WPI, which was due to an increase of the β-sheet content relative to the native state of the proteins [62].

These results confirmed the presence of amyloid fibrils in the solutions of WPI treated with βME, where this effective reductant agent of disulfide bonds opened the tertiary structure of globular proteins changing whey proteins from their native states to an amyloid-like structure [55]. The formation of new extended structures with strong intermolecular and disulfide covalent bonds owing to the unfolding of whey proteins by the reduction of the disulfide bonds generated to a membrane of WPI amyloids with higher affinity for heavy metals compared to a membrane of WPI without the addition of βME [24]. These results were in agreement with the findings of Kabay et al. [63]. These authors prepared a protein membrane of BSA by electrospinning using βME in the electrospun solution to reduce its disulfide bonds breaking its tertiary structure enhancing its supportive properties, thus making the BSA solution spinnable as a natural polymer without the use of a co-polymer.

#### 3.2.4. Contact Angle

**Figure 5:**
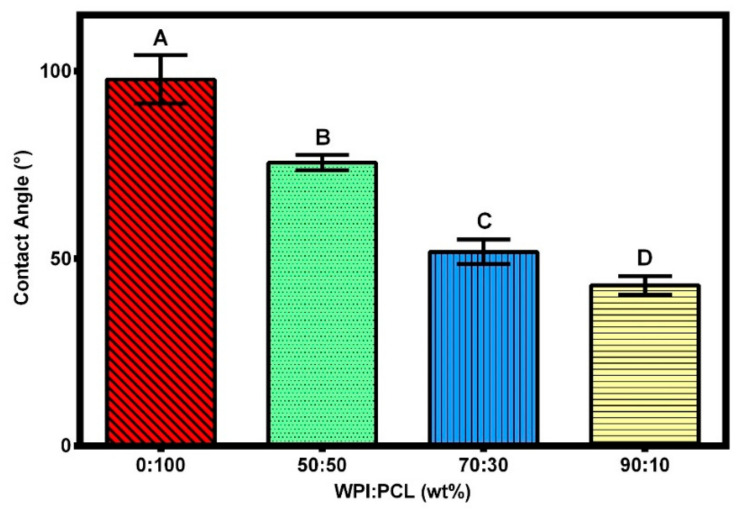
Contact angle of the hybrid membrane of PCL-WPI prepared with different compositions of WPI:PCL (wt%). Error bars are standard deviations (n = 3). Different letters indicate significant differences between different treatments at *p*-level = 0.05 based on the least significant difference (LSD) test.

**Figure 6:**
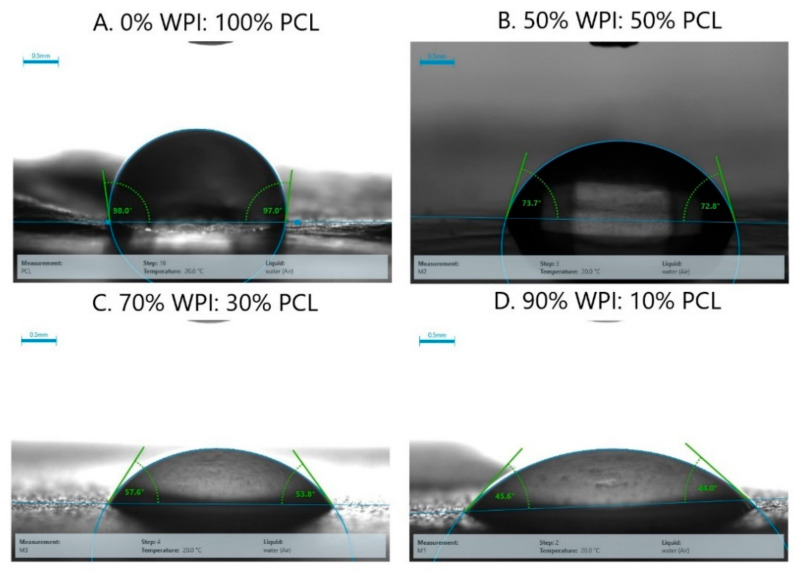
Pictures of the contact angle of the hybrid membrane of PCL-WPI prepared with different compositions of WPI:PCL (wt%).

The effect of hydrophilicity or hydrophobicity on the membrane surfaces was analyzed using contact angle measurements via changing the proportion of WPI and PCL on the spinning solution. The contact angle of the adsorptive membranes is shown in Figure 5 and Figure 6, where the contact angle of the membrane of PCL alone presented a contact angle greater than 90° demonstrating its hydrophobic surface and low wettability in accordance with Lin and Razali [64]. On the other hand, the membranes with 50:50, 70:30, and 90:10 had a contact angle of 75°, 51°, and 42°, respectively. They presented a contact angle below 90° indicating that the membranes had a hydrophilic surface that increased with an increment of WPI on the membrane due to the polarity on the membrane surface produced by the presence of hydroxyl and amide groups on the surface [60].

To produce a suitable adsorptive membrane for membrane filtration, it is important to have an equilibrium between the hydrophilicity and hydrophobicity on the surface of the membrane. Therefore, hydrophilic surfaces are desired because the contact of the membrane with the aqueous solution is facilitated at a smaller contact angle and, in consequence, higher adsorption capacities to remove heavy metals are obtained [65]. Moreover, the presence of hydrophilic groups accelerates the permeation of water molecules through the membrane [66]. However, a super hydrophilic membrane with a contact angle lower than 20° is not desired because it renders the membrane unstable due to its solubility in water [67]. A hydrophobic membrane such as a membrane of PCL alone is not desired because it is well known that the adsorption capacity is not favorable due to the low contact between the water molecules and the membrane; hence, it challenges the affinity of heavy metals with the membrane [65]. These results demonstrated that the membrane obtained at the best results showed a hydrophilic character thus being suitable for heavy metal removal.

#### 3.2.5. Thermogravimetric Analysis

**Figure 7:**
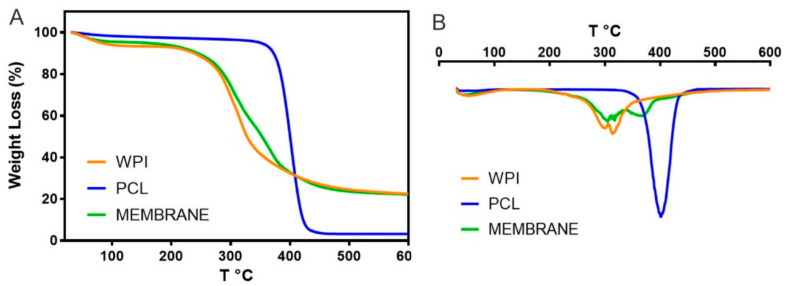
Thermogravimetric (**A**) and Derivate thermogravimetric (**B**) of the pure PCL, pure WPI, and the adsorptive membrane.

Thermogravimetric analysis (TGA) was carried out to determine the degradation temperature of the membrane compared to its main components. This analysis provided information about the weight loss and thermal profile of the adsorptive membrane, PCL and WPI, see Figure 7. The thermal degradation of PCL occurred in one step between 327–450 °C where two types of reactions took place: the random chain scission and unzipping from hydroxyl leading to the formation of ɛ-caprolactone [20,68]. The DTG curve of PCL showed that the degradation temperature of PCL was at 400 °C with a weight loss of 70%, and almost complete degradation occurred at 460 °C with a weight loss of 96%, which is in agreement with Seyedsalehi et al. [69].

TGA analysis showed that the mass loss of the WPI and the membrane occurred in two and three stages, respectively. The first weight stage below 200 °C was due to the evaporation of moisture in WPI and the membrane sample, and the water loss weight percentages were 4 and 6%, respectively. The second decomposition peak corresponded to the main thermal degradation zone with about 30–40% of the weight loss between 290–310 °C and 300–315 °C for WPI and the membrane, respectively, which could be attributed to the breakage of peptide bonds and the decomposition of molecular amine units of WPI thus generating an exothermic change in the DTA [70]. The membrane presented a third decomposition peak at 361 °C due to the decomposition of the PCL of the membrane into its monomers of ɛ-caprolactone. Finally, the membrane presented degradation temperatures similar to the WPI because it was composed of 88% WPI, but there was a low increment in the degradation temperatures due to the composition of PCL into the membrane. The third degradation stage was confirmed by the mass loss percentage of the PCL degradation. In this way, it was important to know the degradation behavior of the membrane due to temperature was an important variable for carrying out the adsorption process, and could be a relevant factor when choosing a material to produce a filtration membrane in large-scale applications

### 3.3. Chromium Adsorption

#### 3.3.1. Kinetics

**Figure 8:**
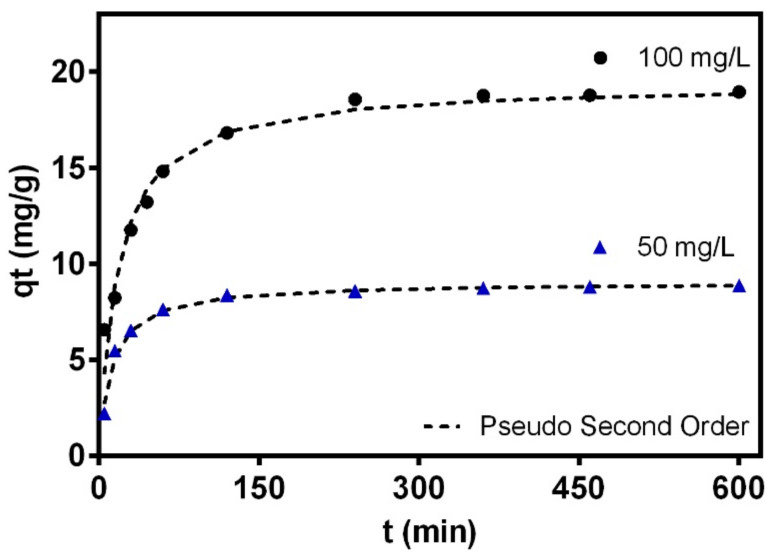
Kinetics for the chromium adsorption on the hybrid membrane at initial concentrations of 50 ppm and 100 ppm, 30 °C and pH 2.

The adsorption kinetics were studied to evaluate the adsorption rate between the surface of the hybrid membrane of PCL-WPI and Cr ions in the solution at concentrations of 50 and 100 mg/L. Figure 8 shows that the hybrid membrane adsorbed a large amount of Cr in about 60 min, which could be due to the presence of many adsorption sites on the adsorbent surface [17]. However, the adsorption of Cr decreased over time by the occupation of the active sites of the membrane and the adsorption equilibrium was achieved after 3.5 h.

These experimental data were fitted using the kinetics classic models of pseudo-first, pseudo-second-order and Intra-particle diffusion. Note that the first-order kinetic model is commonly used to model the first adsorption step at the initial period and the second-order kinetic model is suitable to describe the whole adsorption process involving two stages for the pollutant removal where the first one was fast and reached the equilibrium quickly, the second was slower, and continued for a long contact time [71]. The intraparticle diffusion model involves three steps, which consist in a film diffusion, the intraparticle diffusion and the attachment of the adsorbate that occurs rapidly and it is not a rate limiting step. The pseudo-second-order model was the best to fit the experimental data with R^2^ > 0.97 with modeling errors lower than 9%, see Table 6. Thus, it can be concluded that the rate of the adsorption process relied on two adsorption processes where Cr(VI) ions were adsorbed on the surface of the membrane and also possibly reduced to Cr(III) [72]. Considering that pseudo-second order represented the adsorption process better on the hybrid membrane, this suggests that chemisorption may be the rate controlling mechanism [73]. Moreover, the low R^2^ value of the intraparticle diffusion model was evidence to conclude that the diffusion occurred relatively quickly from the solution, and Cr adsorption on the hybrid membrane of WPI-PCL did not follow this model. Therefore, it could be expected that the adsorption may be an external surface process in the absence of internal diffusion [74].

The chromium removal rates ranged from 0.002 and 0.01 mg/min thus indicating the pollutant diffusion rate from the liquid phase to the outer surface of the hybrid membrane. The increment of the adsorption capacity concerning the initial concentration of Cr confirmed that the driving force of the concentration can overcome the mass transfer resistance of Cr ions between the aqueous and solid phases [75].

#### 3.3.2. Isotherms

**Figure 9:**
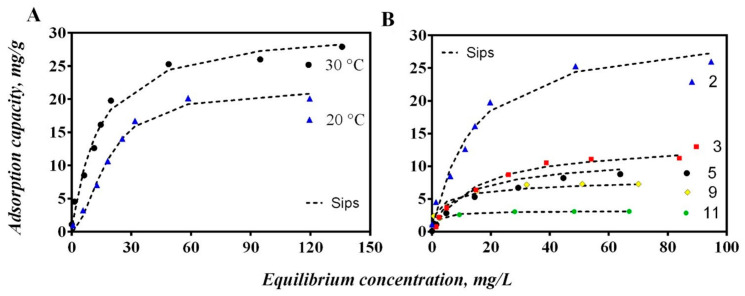
Isotherms for chromium adsorption from aqueous solutions using an adsorptive membrane of WPI-PCL. (**A**) Effect of temperature at pH 2 and (**B**) effect of pH at 30 °C.

The chromium adsorption isotherms using the hybrid membrane of WPI-PCL at different temperature and pH are reported in Figure 9.

The adsorption capacity increased significantly with the decrease of solution pH from 3 to 2 and reached a maximum value of 31 mg/g at pH 2, see Figure 9B. This could be explained by the fact that the protonation of the membrane of WPI-PCL was enhanced at highly acidic conditions. Thus, when the pH of the solution decreased below to the PZC of the membrane that was pH 3, the surface was positively charged by the protonation of amino groups of the WPI proteins, which resulted in positively charged surfaces favoring the adsorption of anionic species such as HCrO_4_^−^ [76]. The same behavior was observed by Jin et al. [77]. These authors prepared a keratin/PET nanofiber membrane with a PZC at pH 3.77, thus indicating that the surface of K-PET-5 was positively charged at pH 3 and attracted anions such as HCrO_4_^−^. It is important to remark that PCL alone did not present affinity by heavy metal ions due to its hydrophobic character and even when it has a carbonyl group that could be protonable at low pH, it did not present affinity by Cr or another heavy metal [78].

Adsorption equilibrium experiments were performed at different temperatures to analyze the Cr removal, see Figure 9A. The results demonstrated that the removal performance of the hybrid membrane of WPI increased with the increase of temperature obtaining the maximum adsorption capacity at 30 °C and thus confirming the temperature-dependent nature of this adsorption process [79]. This behavior demonstrated that the adsorption process was endothermic where the estimated enthalpy was 45.45 kJ/mol, see Table 7. These results indicated that the adsorption process of this membrane to remove Cr could be attributed to a physico-chemical adsorption instead of a purely physical or chemical adsorption process [80]. As stated, the functional groups of the whey proteins in the membrane interacted with Cr ions through electrostatic interactions and coordinated bonds [47]. The Gibbs free energy for the Cr adsorption on the hybrid membrane of WPI-PCL was negative (see Table 7), which indicated that the adsorption process was spontaneous [81]. The entropy was positive indicating the increase of randomness mainly on Cr adsorption at the solid/solution interface during the separation process where ion replacement reactions occurred [80].

The experimental adsorption isotherms were correlated with classical isotherm equations such as Langmuir, Freundlich and Sips. Results of this modeling analysis are reported in Table 8 and Table S3. Sips equation was the best model for fitting the Cr isotherms of the WPI-PCL hybrid membrane with R^2^ > 0.96 and modeling errors lower than 7%. Sips isotherm is a model that includes the features of Langmuir and Freundlich models. It reduces to the Freundlich isotherm at low concentrations and predicts a monolayer adsorption capacity characteristic of the Langmuir isotherm at higher concentrations [82].

The heterogeneity factor (n_s_) of Sips model indicates the heterogeneity of the surface sites that carry out the adsorption process. It indicates a homogeneous surface if its value is equal to 1, the Sips model simplifies to the Langmuir model, while a significant difference from 1 indicates a heterogenous surface [83]. Results of this parameter may suggest that Cr ions were weakly bonded to the surface of the adsorbent thus confirming the electrostatic interactions between the adsorbent and adsorbate and also the presence of different binding sites on the adsorbent surface [84]. The K_L_ parameter indicates the level of interaction between the adsorbate and adsorbent, where a value between 0 and 1 describes favorable adsorption of Cr onto the hybrid membrane [85].

#### 3.3.3. Mechanism

**Figure 10:**
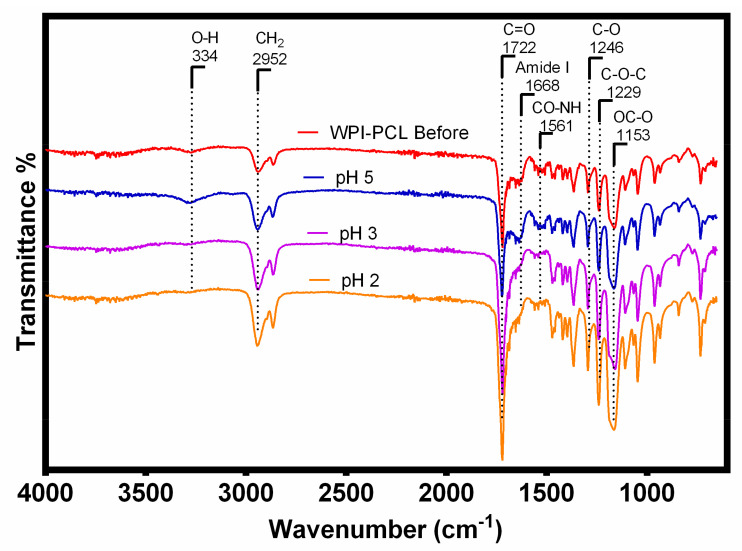
FTIR spectra of the WPI-PCL hybrid membrane before and after the chromium adsorption at pH 5, 3 and 2.

**Figure 11:**
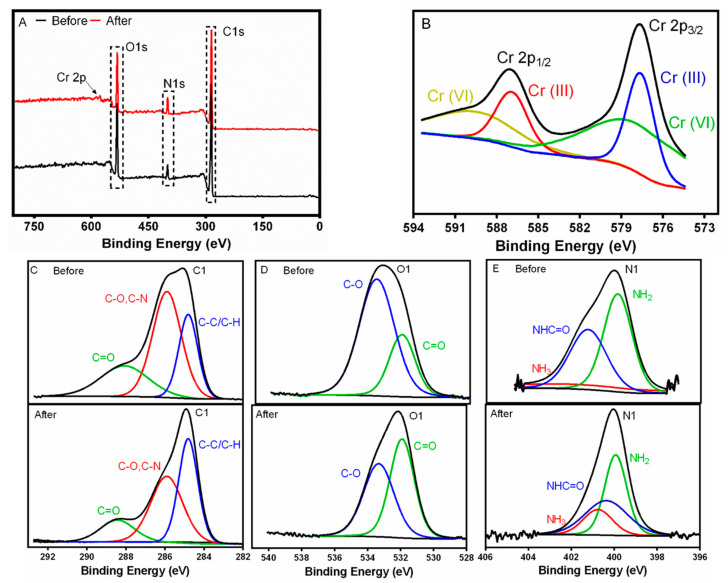
XPS analysis of the WPI-PCL hybrid membrane before and after chromium adsorption. (**A**) Survey spectra, (**B**) Cr high-resolution XPS spectra after adsorption, (**C**) C 1s, (**D**) O 1s and (**E**) N 1s before and after adsorption.

To gain further insight into the possible Cr adsorption mechanism, FTIR and XPS analyses of the membrane after the metal adsorption were performed. FTIR spectrum (see Figure 10) showed the changes in the functional groups of the membrane before and after the adsorption of Cr at pH 5, 3 and 2. FTIR spectra of the membranes after adsorption at pH 5 did not show significant differences compared with the membrane before the adsorption due to the low quantity of Cr loaded on the sample surface. However, the membrane after the Cr adsorption at pH 2 and 3 showed that the bands related to the amides at 1668 and 1561 cm^−1^ had a lower intensity and even disappeared in comparison with the raw membrane, which indicated that the amide groups were involved in this adsorption process. Considering that the membrane was under acidic conditions, the possible adsorption mechanism could be due to the amide bonds of this membrane being hydrolyzed into carboxyl and amino groups, which also explained the increase in the intensity of the carbonyl group at 1722 cm^−1^ [77]. Moreover, at pH 2 and 3 the amino group of the proteins could be protonated into NH_3_ favoring the electrostatic interaction with HCrO_4_^−^, as stated by Jin et al. [77]. The same was confirmed by XPS in Figure 11E with the appearance of NH_3_ peak after the adsorption of Cr.

The XPS results (see Figure 11A) of the membrane display peaks of O 1s, C 1s and N 1s. The membrane obtained after adsorption showed a new peak at 577 eV of Cr 2p, which was subdivided in two different peaks belonging to Cr(VI) and Cr(III) species [86]. The high-resolution Cr 2p XPS (see Figure 11B) spectra showed the typical two broad peaks at 587.4 and 577.9 eV, which were assigned to Cr 2p1/2 and Cr 2p3/2, respectively. The peak of Cr 2p1/2 could be deconvoluted into two peaks with binding energies of 589.6 and 586.9 eV for Cr(III) and Cr(VI), respectively [87]. Likewise, the Cr 2p3/2 XPS spectrum was deconvoluted into two peaks of Cr(III) and Cr(VI) species at 579.3 and 577.6 eV, which indicated the coexistence of Cr(VI) and Cr(III) species. This evidence confirmed that the membrane reduced partially Cr(VI) into Cr(III) [88].

The high-resolution C 1s, O 1s and N1s XPS spectra were also analyzed (see Figure 11C–E. The C 1s spectrum of the membrane was deconvoluted into C=O at 288.7 eV, C-O/C-N at 286.1 and C-C/C-H at 284.8 eV. The relative content of C-C/C-H decreased after the Cr adsorption and the relative content of C-O increased in the membrane (see Table S4). These results may indicate that carbon in the membrane could be oxidized by Cr(VI) and Cr may be reduced to Cr(III) as explained by Yuan et al. [87]. Moreover, O1s spectrum was deconvoluted into C-O at 533.6 eV and C=O at 532.1 eV, and a decrease in the binding energy of O 1s after Cr adsorption was observed and, as a result, it increased the electron density. This finding may indicate a complexation between O and Cr, which was also confirmed by the increase in C=O group and the decrease of C-O group (see Table S4). As stated by Rodzik et al. [47], the proteins interact with metal ions and metalloproteins are formed by coordination bonds between metal ions and functional groups of amino acids, such as carboxyl [47]. Finally, a decrease in the concentration of nitrogen was observed (see Table S5) and the concentration of NH_3_ increased in the membrane after the adsorption confirming the electrostatic interaction with HCrO_4_^−^, in agreement with FTIR results.

#### 3.3.4. Comparative Study

Table 9 shows a comparison of the adsorption properties of the hybrid membrane obtained in this study with other hybrid membranes obtained by electrospinning reported in the literature. The adsorption capacity of WPI-PCL hybrid membrane was highly competitive compared to the membrane of zein/nylon-6 nanofiber membrane [16], PVDF/silica thiol nanofiber membrane [89], PCL/Clay electrospun fibers [20], cerium oxide nanoparticles embedded in polyacrylonitrile [90] and citric-acid-incorporated cellulose mats [91], which presented adsorption capacities of 4.73, 15.1, 24.57, 28.09 and 20 mg/g. Some of these studies have affirmed that the low Cr removal was due to the high amount of low-affinity polymers in the fibers such as PVA [20].

In addition, there are competitive membranes made by electrospinning reported in the literature such as ZIF-8@ZIF-8/polyacrylonitrile nanofibers [92], keratin/PET nanofiber membrane [77], and polyacrylonitrile/ionic covalent organic framework nanofibers [14] that presented adsorption capacities of 39.68, 75 mg/g and 173 mg/g. In addition, the higher performance of keratin/PET nanofiber membrane could be due to the use of a polymer such as PET that alone showed an adsorption capacity of 27.27 mg/g, but it is well known that PET biodegrades at a slower rate than PCL [94]. It is important to note that the equilibrium time depends on agitation speed, particle size and temperature so that these parameters can be modified to reduce the equilibrium time of the membrane regarding the application.

## 4. Conclusions

In this work, a WPI-PCL hybrid membrane was prepared by electrospinning and applied to adsorb Cr from an aqueous solution. The effect of the collector distance and composition of βME, WPI, and PCL on the adsorption capacity of this hybrid membrane was studied, thus demonstrating that the addition of βME allowed the functionalization of PCL with amyloid-type WPI proteins with high affinity to heavy metal ions. SEM characterization of the hybrid membrane showed a fiber morphology with ultrathin fibers with an average fiber diameter of 62.2 nm, without the presence of beads. The presence of amyloid-type proteins and the functional groups of WPI and PCL in the electrospun fibers were confirmed by FTIR spectra and Red Congo assay, respectively. The hydrophilic nature of the membrane was confirmed by the contact angle measurement, proving that the hydrophilic surface of the membrane enhanced the affinity of Cr ions by the hydroxyl and amide groups of WPI. TGA analysis allowed us to conclude that the hybrid membrane presented degradation temperatures similar to WPI because its WPI composition was 88%, but also presented a low increment on the degradation temperatures due to the PCL composition. These characteristics could be relevant for further applications.

The adsorption capacities of the hybrid membrane increased with an increase in temperature showing an endothermic adsorption process. The best performance of this membrane was obtained at strong acidic conditions (pH 2) demonstrating that its PZC had a strong influence on heavy metal removal by electrostatic interactions. Adsorption experiments indicated that the hybrid membrane exhibited a reasonably high adsorption performance to remove Cr ions from aqueous solutions with a good equilibrium time compared with other membranes made by electrospinning and reported in the literature. FTIR and XPS analyses confirmed that Cr(VI) was reduced to Cr(III) by the membrane under acidic conditions and the Cr removal could be due to a combination of electrostatic interactions, redox reactions, and the formation of metallocomplexes. This work provides a novel method to fabricate a hybrid membrane with an amyloid-type fibril made of WPI and PCL, with notable characteristics to be used as potential material to remove heavy metals from water. This material holds promise for application in membrane technology at industrial scale.

## Figures and Tables

**Table 1:** Central Composite Design (CCD) to prepare the WPI/PCL hybrid adsorbent membrane for chromium removal.

Variable	Unit	Factors	Levels		
			−1	0	1
WPI	%	A	50	70	90
βME	%	B	0.1	0.65	1.2
Collector Distance	cm	C	7	10	13

α = 1 (star or axial point for orthogonal small CCD in the case of three independent variables) with 5 center points.

**Table 2:** Central Composite Design (CCD), experimental and analytical response for the preparation of WPI/PCL hybrid membrane. The factors were A-WPI (%), B-βME (%) and C, Collector distance (cm).

Run	A	B	C	*qe* (mg/g)	Diameter (nm)	ST (mN/m)	Viscosity (Pa·s)	Beads
1	50	0.1	7	1.6	413	30.2	0.383	NO
2	70	1.2	10	1.44	52	30.8	0.048	YES
3	70	0.65	10	0.6	186	30.5	0.061	NO
4	70	0.65	10	1.02	188	30.6	0.057	NO
5	70	0.65	13	0.69	336	30.7	0.057	NO
6	50	0.65	10	0.81	350	30.5	0.323	NO
7	50	1.2	13	0.92	528	30.9	0.194	NO
8	70	0.1	10	0.45	299	30.3	0.059	YES
9	70	0.65	10	0.88	180	30.6	0.056	NO
10	70	0.65	10	1.42	170	30.5	0.050	NO
11	70	0.65	7	2.12	160	30.6	0.053	YES
12	90	0.1	13	1.56	68	30.7	0.007	YES
13	90	1.2	7	2.74	53	31.3	0.006	YES
14	90	0.65	10	1.83	48	31	0.005	YES
15	70	0.65	10	0.92	175	30.7	0.059	NO

**Table 3:** Results of ANOVA for the variables analyzed in the optimization design to prepare WPI/PCL hybrid membrane.

	*qe* (mg/g)			Diameter (nm)			ST (mN/m)			Viscosity (Pa·s)		
	SS	df	*p*-Value	SS	df	*p*-Value	SS	df	*p*-Value	SS	df	*p*-Value
Model	5.22	9	0.0162	288,599	9	0.0001	1.03	9	0.0042	0.18	9	0.0001
A	0.52	1	0.0422	45,602	1	0.0001	0.10	1	0.0149	0.05	1	0.0001
B	0.49	1	0.0455	30,505	1	0.0001	0.15	1	0.0065	0.00	1	0.1856
C	1.02	1	0.0126	15,488	1	0.0001	0.01	1	0.3084	0.00	1	0.5547
AB	0.08	1	0.3239	4107	1	0.0002	0.00	1	0.6927	0.00	1	0.0001
AC	0.18	1	0.1644	29,403	1	0.0001	0.00	1	0.4217	0.00	1	0.0002
BC	0.00	1	0.7908	3888	1	0.0002	0.00	1	0.8538	0.00	1	0.0099
A^2^	0.30	1	0.0939	1058	1	0.0048	0.05	1	0.0393	0.03	1	0.0001
B^2^	0.00	1	0.8376	30	1	0.4542	0.00	1	0.4108	0.00	1	0.0578
C^2^	0.47	1	0.0483	12,494	1	0.0001	0.01	1	0.2815	0.00	1	0.114
Lack of Fit	0.01	1	0.9408	3	1	0.8387	0.00	1	0.7419	52	1	0.173
Pure Error	0.35	4		225	4		0.03	4		75	4	
R^2^	0.94			0.99			0.96			0.99		
R^2^ Adj	0.82			0.99			0.9			0.99		

**Table 4:** Best preparation conditions to produce WPI/PCL hybrid membrane based on the results of adsorption capacity, fiber diameter, viscosity, and surface tension. Validated values were expressed as mean ± SD of triplicate.

Optimal Preparation Conditions	
WPI (%)	88
2-mercaptoethanol (%)	1.2
Collector Distance (cm)	7.1
q (mg/g) predicted	2.73
q (mg/g) validated	2.59 ± 0.21
Error (%)	5.0
Diameter predicted (nm)	39.1
Diameter Validated (nm)	38.3 ± 2.3
Error (%)	2.2
Viscosity predicted (cP)	5.9
Viscosity validated (cP)	5.7 ± 0.4
Error (%)	4.3
Surface Tension predicted (mN/m)	31.2
Surface Tension validated (mN/m)	30.5 ± 0.1
Error (%)	2.4

**Table 5:** Effect of the total solids on the adsorption capacity, fibril diameter and bead formation of WPI/PCL hybrid membrane.

Total Solids %	*qe* (mg/g)	Diameter (nm)	Viscosity (Pa·s)	Surface Tension (mN/m)	Beads Formation
15%	2.4	36.2	0.008	30.3	YES
18%	8.61	62.2	0.034	30.5	NO
21%	-	-	0.089	30.7	-

All values are presented as means (*n* = 3).

**Table 6:** Adsorption kinetic rates for chromium removal from aqueous solution using the hybrid membrane of WPI-PCL. Experimental conditions: pH 2 and 30 °C.

Model	Parameters	50 mg/L	100 mg/L
Pseudo First Order	q_te_ (mg/g)	8.57	18.34
	K1 (mg/min)	0.05	0.03
	R^2^	0.97	0.94
Pseudo Second Order	q_te_ (mg/g)	9.03	19.40
	K2 (mg/min)	0.01	0.002
	R^2^	0.99	0.97
Intraparticle diffusion	Kd (mg/(g min^1/2^)	0.10	0.33
	C (mg/g)	5.38	10.30
	R^2^	0.49	0.63

**Table 7:** Thermodynamic parameters for the chromium adsorption on the hybrid membrane of PCL and WPI.

T (°C)	Δ*G*°, kJ/mol	ΔH°, kJ/mol	ΔS°, kJ/mol K
20	−2.17	45.45	0.16
30	−3.80		

**Table 8:** Results of data correlation of chromium adsorption isotherms of WPI-PCL adsorptive membrane using the Sips model at pH 5.

Temperature (°C)	pH	Sips			
		q_s_ (mg/g)	Ks (L^ns^/mg^ns^)	ns	R^2^
20	2	21.5	0.056	1.8	0.99
30	2	31.0	0.074	1.0	0.98
	3	13.2	0.074	1.0	0.99
	5	12.3	0.077	0.7	0.98
	9	9.0	0.220	0.5	0.96
	11	3.1	0.580	1.0	0.99

**Table 9:** Comparison of adsorption capacities of different membrane-based adsorbents for the chromium removal.

Adsorbent	pH	Ci (mg/L)	*qe* (mg/g)	Equilibrium Time	Reference
Zein/nylon-6 nanofiber membrane	2	5–25	4.73	60 min	[16]
Polyacrylonitrile/ionic covalent organic framework nanofibers	3	1–1000	173	4 h	[14]
PCL/Clay and PVA/Clay Electrospun Fibers	5	200	24.57	-	[20]
Cerium oxide Nanoparticles embedded in polyacrylonitrile nanofiber	6	20	28.09	3 h	[90]
Cellulose acetate (CA), acetone/DMAc, Citric Acid	2	20–60	20	2 h	[91]
ZIF-8@ZIF-8/polyacrylonitrile nanofibers	2	1–500	39.68	90 min	[92]
PAN-GO-Fe_3_O_4_ hybrid nanofibers	3	50	124.3	70 min	[93]
keratin/PET nanofiber	3	20–120	75	4 h	[77]
PVDF/silica thiol nanofiber	2	31	15.1	60 min	[89]
Hybrid membrane of WPI-PCL	2	10 to 300	31.0	3.5 h	This study

## Data Availability

Not applicable.

## Supplementary Materials

The following data are available online at https://www.mdpi.com/article/10.3390/nano12162744/s1, Table S1: Classical kinetic models, Table S2: Classical isotherm models, Table S3: Results of data correlation of adsorption isotherms to classical models for chromium removal from aqueous solutions using and WPI-PCL adsorptive membrane, Table S4: Contributions of individual chemical moieties in the high-resolution spectra of the WPI-PCL adsorptive membrane before and after chromium adsorption, Table S5: Results of the atomic concentration of the general XPS before and after chromium adsorption by the membrane.
