# Supplementary material for: Preparation and Characterization of an Electrospun Whey Protein/Polycaprolactone Nanofiber Membrane for Chromium Removal from Water
Source: Nanomaterials (Basel). 2022 Aug 10;12(16):2744. doi: 10.3390/nano12162744 (PMC9413122; doi:10.3390/nano12162744)
Supplement: Supplementary file 1 [file nanomaterials-12-02744-s001.zip › nanomaterials-1852376-supplementary.pdf]

Preparation and Characterization of an Electrospun Whey Protein/Polycaprolactone Nanofiber Membrane for Chromium Removal from Water

Laura Cristina Ramírez-Rodríguez [1], María Ximena Quintanilla-Carvajal [2], Didilia Ileana Mendoza-Castillo [3,4], Adrián Bonilla-Petriciolet [4] and Carlos Jiménez-Junca [5,*]

[1] Maestría en Diseño y Gestión de Procesos Facultad de Ingeniería, Campus Universitario Puente del Común, Universidad de la Sabana, Km. 7 Autopista Norte, Chia 25001, Colombia
[2] Agroindustrial Processes Research Group, Campus Universitario Puente del Común, Universidad de La Sabana, Km. 7 Autopista Norte, Chia 25001, Colombia
[3] CONACYT, Ciudad de México 03940, Mexico
[4] Departamento de Ingeniería Química, Instituto Tecnológico de Aguascalientes, Aguascalientes 20256, Mexico
[5] Bioprospecting Research Group, Campus Universitario Puente del Común, Universidad de La Sabana, Km. 7 Autopista Norte, Chia 25001, Colombia
* Correspondence: carlos.jimenez@unisabana.edu.co; Tel.: +57-861-5555-25010

Table S1. Classical kinetic models.

Model	Equation
Pseudo first order	$q_t = q_{te}(1 - e^{-K_1 t})$
Pseudo Second order	$q_t = \frac{q_{te}^2 K_2 t}{1 + q_{te} K_2 t}$
Intraparticle diffusion	$q_t = k_{te} t^{0.5} + C$

Where q_t (mg/g) and q_{te} (mg/g) are the quantity of chromium adsorbed per mass unit of membrane at a given time t, and at equilibrium, respectively; K_1 and K_2 (mg min^{-1}) are the first-order rate constant and second-order rate constant, respectively; k_{te} (mg/(g $min^{1/2}$) is the Intraparticle diffusion rate constant; C is a model constant (mg/g).

Table S2. Classical isotherm models.

Model	Equation
Langmuir	$q_e = \frac{q_m K_L C_e}{1 + K_L C_e}$
Freundlich	$q_e = K_F C_e^{1/n}$
Sips	$q_e = \frac{q_s K_s C_e^{n_s}}{1 + K_s C_e^{n_s}}$

Where q_e and C_e are the adsorption capacity (mg/g) and concentration (mg/L) at equilibrium, q_m and q_s are the theoretical maximum adsorption capacity (mg/g), K_L (L/mg), K_F ($mg^{1-1/n}$ $L^{1/n}$ g^{-1}), Ks (L^{ns}/mg^{ns}) represents the Langmuir, Freundlich and Sips equilibrium constants, n is the parameters characteristic of the adsorbent-adsorbate system and n_s is the heterogeneity Sips parameter.

Table S3. Results of data correlation of adsorption isotherms to classical models for chromium removal from aqueous solutions using and WPI-PCL adsorptive membrane.

Temperature (°C)	pH	Model					
		Langmuir			Freundlich		
		q_m	K_L	R^2	K_F	n	R^2
20	2	26.4	0.039	0.96	3.1	2.4	0.87
30	2	31.4	0.072	0.98	5.7	2.8	0.94
	3	13.5	0.069	0.98	2.0	2.4	0.92
	5	10.5	0.110	0.98	2.3	2.8	0.96
	9	7.5	0.327	0.96	3.2	5.3	0.92

	11	3.1	0.561	0.99	1.4	5.1	0.86

Table S4. Contributions of individual chemical moieties in the high-resolution spectra of the WPI-PCL adsorptive membrane before and after chromium adsorption.

**Element**	**Chemical moiety**	**Before**		**After**	
		Binding Energy	% Relative Content	Binding Energy	% Relative Content
Cr 2p 3/2	Cr (VI)	-	-	577.6	23.73
	Cr (III)	-	-	579.3	41.25
Cr 2p 1/2	Cr (VI)	-	-	586.89	14.98
	Cr (III)	-	-	589.56	20.04
C1 s	C–(C,H)	284.8	31.92	284.81	41.07
	C–O	286.1	49.71	285.9	43.49
	C=O	288.73	18.37	288.43	15.45
O1 s	C–O	533.66	71.88	533.32	48.1
	C=O	532.13	28.12	531.91	51.9
N1 s	NHC=O	401.26	38.17	400.36	36.83
	NH2	399.87	48.07	399.93	44.43
	NH3	401.91	13.76	400.75	18.75

Table S5. Results of the atomic concentration of the general XPS before and after chromium adsorption by the membrane**.**

**Atomic concentration [%]**		
**Element**	**Before**	After
C	74.95	75.07
O	18.18	19.8
N	6.86	4.37
Cr	-	0.76
